# Voluntary consensus based geospatial data standards for the global illegal trade in wild fauna and flora

**DOI:** 10.1038/s41597-022-01371-w

**Published:** 2022-06-03

**Authors:** Meredith L. Gore, Lee R. Schwartz, Kofi Amponsah-Mensah, Emily Barbee, Susan Canney, Maria Carbo-Penche, Drew Cronin, Rowan Hilend, Melinda Laituri, David Luna, Faith Maina, Christian Mey, Kathleena Mumford, Robinson Mugo, Redempta Nduguta, Christopher Nyce, John McEvoy, William McShea, Angelo Mandimbihasina, Nick Salafsky, David Smetana, Alexander Tait, Tim Wittig, Dawn Wright, Leah Wanambwa Naess

**Affiliations:** 1 Department of Geographical Sciences, University of Maryland, College Park, MD USA; 2 Office of the Geographer and Global Issues, US Department of State, Washington, DC USA; 3 Centre for Biodiversity Conservation Research, University of Ghana, Accra, Ghana; 4 Department of Operations Management, University of Alabama, Tuscaloosa, AL USA; 5 Department of Zoology, University of Oxford, Oxford, United Kingdom; 6 Wildlife Conservation Society South Sudan Program, Juba, South Sudan; 7 International Conservation, North Carolina Zoo, Asheboro, NC USA; 8 Department of Supply Chain Management, Michigan State University, East Lansing, MI USA; 9 Department of Ecosystem Science and Sustainability, Colorado State University, Fort Collins, CO USA; 10 Luna Global Networks & Convergence Strategies, Washington, DC USA; 11 Space for Giants, Nanyuki, Kenya; 12 Special Wildlife Protection Fund, Ministry of Forestry and Wildlife, Yaoundé, Cameroon; 13 Booze Allen Hamilton, McLean, VA USA; 14 Regional Centre for Mapping of Resources for Development (RCMRD), Nairobi, Kenya; 15 Embassy Juba, US Department of State, Juba, South Sudan; 16 Smithsonian Conservation Biology Institute, Front Royal, VA USA; 17 Madagascar Program Patrol Coordinator, Durrell Wildlife Conservation Trust, Antananarivo, Madagascar; 18 Foundations of Success, Washington, DC USA; 19 Environmental Systems Research Institute (ESRI), Ottawa, Canada; 20 National Geographic Society, Washington, DC USA; 21 United for Wildlife Taskforce, London, United Kingdom; 22 Focused Conservation, Flemming Island, FL USA; 23 Environmental Systems Research Institute (ESRI), Redlands, CA USA; 24 Department of Agriculture, Rural Development Blue Economy, and Sustainable Environment, African Union Commission, Addis Ababa, Ethiopia

**Keywords:** Environmental impact, Geography, Databases

## Abstract

We have more data about wildlife trafficking than ever before, but it remains underutilized for decision-making. Central to effective wildlife trafficking interventions is collection, aggregation, and analysis of data across a range of source, transit, and destination geographies. Many data are geospatial, but these data cannot be effectively accessed or aggregated without appropriate geospatial data standards. Our goal was to create geospatial data standards to help advance efforts to combat wildlife trafficking. We achieved our goal using voluntary, participatory, and engagement-based workshops with diverse and multisectoral stakeholders, online portals, and electronic communication with more than 100 participants on three continents. The standards support data-to-decision efforts in the field, for example indictments of key figures within wildlife trafficking, and disruption of their networks. Geospatial data standards help enable broader utilization of wildlife trafficking data across disciplines and sectors, accelerate aggregation and analysis of data across space and time, advance evidence-based decision making, and reduce wildlife trafficking.

## Introduction

The contemporary illegal trade in wild flora and fauna, known colloquially as illegal wildlife trade (IWT), is a societal problem that is global, species and ecosystem agnostic, and very profitable for professional criminals^1^. IWT was historically tackled by the conservation and sustainable development sectors. Today, IWT is connected to risks associated with national security, financial crime, social media, technology, social conflict, justice and inclusion, urbanization, gender, mass media, human health, and possibly state capture^2–4^.New regulations, public-private partnerships, university courses, documentary movies, and scientific collaborations have emerged in response to the harms associated with IWT^5^. Paralleling the growth in recognition of IWT has been an explosion in IWT-related data. The sources, volume, scope, and scale of data characterizing IWT, its workforce, and counter-IWT efforts have all grown substantially in a matter of years, as have calls for data sharing. Multiple authorities have recognized the importance of data for IWT decision making (e.g., U.S. Fish and Wildlife Service, United Nations Office on Drugs and Crime, African Union Commission, Alvarado Quesada of Costa Rica, Bongo Ondimba of Gabon, Conservation International)^6,7^.

Data aggregation and disaggregation for IWT-related decision-making does not require that data be warehoused together or subject to identical privacy requirements, but it does require interoperability between data sets^8^. Collecting, aggregating, analyzing^1^, and sharing geospatial information supports evidence-based decision making that can, for example, disrupt wildlife trafficking networks, reduce crime rates, or enable strategic prevention of harm^9^.

Many sectors are working to combat IWT worldwide, but often without coordination. Even when there is collaboration, data can be onerous to navigate and aggregate transparently because IWT data are collected for different reasons, by different sectors, and in different countries, ecosystems, languages, and crime spaces. The effects of aggregated IWT data have been largely incremental for decision-making and intervention strategies. Geospatial data standards for combating IWT would help address these shortcomings by providing a common framework upon which any group or platform could build.

No data standards exist for measuring and tackling IWT intervention success, such as disrupting networks. No international authority exists for promoting and propagating such standards. Thus, the full potential of geospatial data to support decisions that combat IWT remains unrealized. Our goal was to create geospatial data standards for combating IWT using established tools such as engagement-based workshops, online tools, and electronic communication with stakeholders willing to participate from multiple sectors. A participatory approach was selected over a top-down approach or expert elicitation because it is more likely to ensure the data standards are accessible or used, nor does it necessarily encourage sharing of data^1^. We do not address the logistics of ethically sharing and using data herein. We address the prerequisite issue of data standards.

Data standards need to be adaptable and, at a minimum, delineate common required elements so that unstructured and structured data can be aggregated across jurisdictions, ecosystems, or countries^10^. Thus, we created voluntary, consensus-based geospatial data standards for the global IWT, as well as an accompanying data dictionary; we did not create a data repository, nor did we identify a validation protocol. The standards were deliberately developed to support the diversity of actors working to combat IWT including researchers, investigators, prosecutors, conservationists, and rangers. The standards were designed to support stakeholders in their reporting requirements, underpin creative research on wildlife trafficking patterns, help uncover links to transnational crime, and design strategies to disrupt wildlife trafficking networks.

## Results

**Fig. 1:**
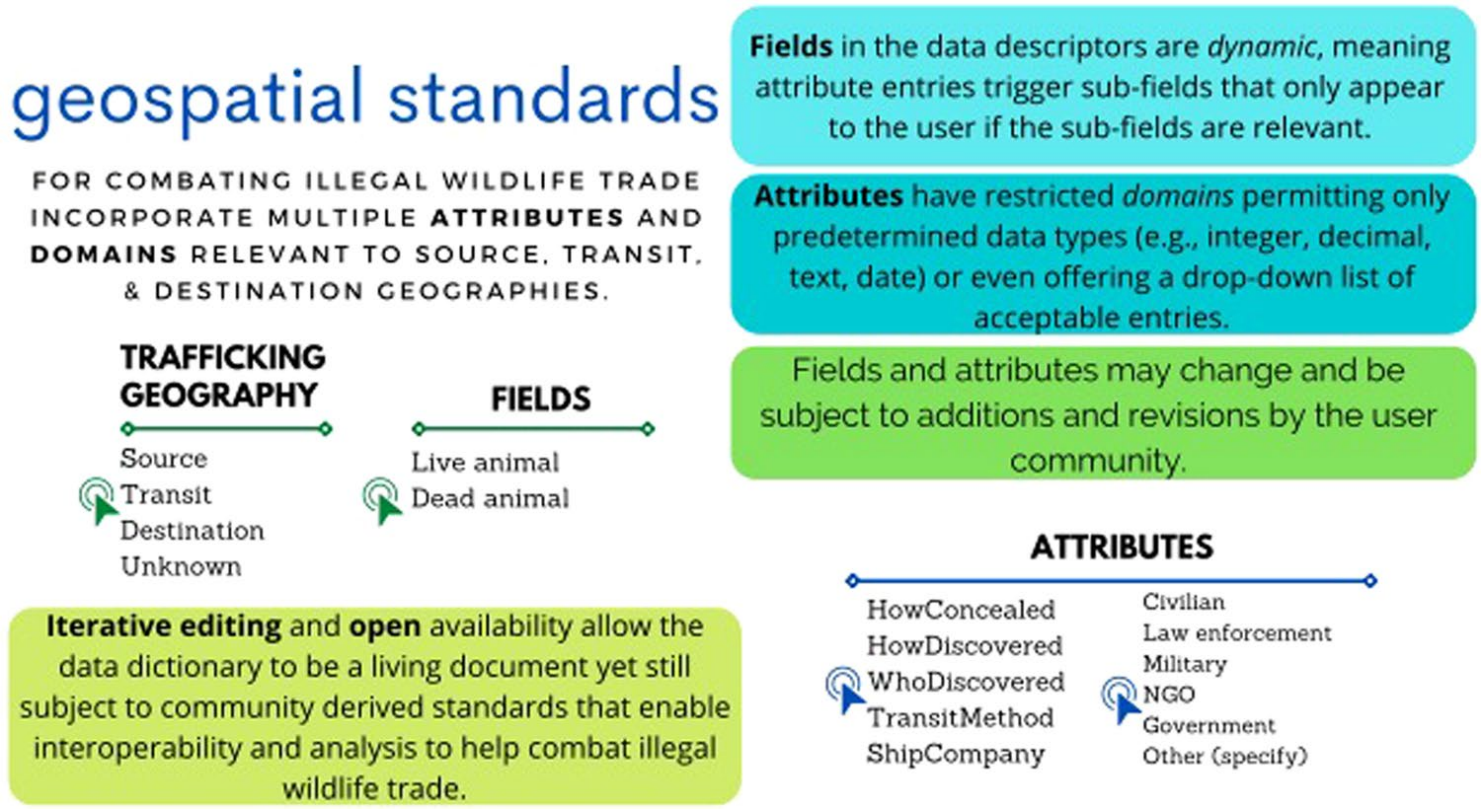
Geospatial data standards for combating wildlife trafficking define many fields and attributes. These data are fundamentally critical to meaningful sharing and analysis of data across physical, digital, political, and organizational boundaries.

Participants emphasized a need for data standards to map onto the “source,” “transit,” and “destination” geographies of wildlife trafficking and be tailored to end users working on preventing and responding to IWT. The format of the standards is species and ecosystem agnostic. The data dictionary focuses on observations, since most baseline (i.e., foundational) and referenced (i.e., thematic) data layers are managed by practitioners operating in realms other than IWT. Participants also agreed that the data dictionary be organized into meta data categories under collective headings (e.g., trafficking geography, Fig. 1, Table 1). Each of these data categories represents a vital source of information for stakeholders combating IWT, which can be captured using dynamic fields and attributes (some with domains) (Fig. 1, Table 1). The remainder of this section describes the primary application of the data categories, fields, attributes, and domains. We provide a description of data categories in the dictionary along with the category’s relevance to the respective disciplines, the category’s fields, attributes, and the attributes’ domains, where applicable.

**Table 1:** The geospatial data standards to combat the global illegal wildlife trade were intentionally derived using interdisciplinary data descriptors to have applicability to the conservation, law enforcement and criminal justice, and supply chain sectors vested in reducing wildlife trafficking.

Geospatial Data Category	Applicability to Conservation Sectors	Applicability to Law Enforcement and Criminal Justice Sectors	Applicability to Supply Chain Sectors
Conservation Information	Understanding wildlife species and populations of interest, conservation status, key risks, degrees of uncertainty.	Understanding targets of harm or risk, which in turn informs the severity of the victimization and informs potential sanctions, penalties, and justice responses.	Understanding market prices can help to define market entry and exit conditions for traffickers.
	Identifying associated human dimensions of conservation, such as market/econometric insight about prices and possibly trend and inference analysis.	Identifying jurisdiction(s) and relevant authorities or agencies that should lead or collaborate.	Identifying species displacement due to uneven enforcement.
			Identifying bottlenecks and choke points where intervention efforts would be most effective for species or population.
	*(geospatial data: point, line, polygon, attribute)*	*(geospatial data: point, polygon, attribute)*	*(geospatial data: point, line, polygon, attribute)*
Criminogenic Information	Understanding which species are hot products for illegal trade and which species might be next.	Understanding efficacy of law enforcement efforts as well as gaps in procedures that lead to failed cases.	Understanding penalties for criminals who are discovered.
		Understanding danger to the public and convergence with other forms of crime (particularly for the presence of weapons).	Determining where enforcement efforts can be increased to harm criminal organizations the most.
		Understanding concealment methods improves detection rates.	Identifying displacement effects and developing coordinated interdiction strategies and network dynamics.
		Understanding where traffickers will be and when helps target enforcement efforts.	
	*(geospatial data: polygon, attribute)*	*(geospatial data: point, polygon, attribute)*	*(geospatial data: point, line, polygon, attribute)*
Data Integrity & Maintenance	Increasing reproducibility of results, data fidelity, reducing assumptions / biases, facilitating associations with other datasets. Allowing others to augment wildlife-related datasets and synthesize across activities.		
	*(geospatial data: point, line, polygon, attribute)*		

The defendants were indicted on five counts of “Wildlife Trafficking in Violation of the Lacey Act.”

### Data descriptors

- Identification Number: a unique identifier that differentiates incidents and links one incident to multiple reports across organizations.
- Discovery: details the illicit products found. Fields include method of discovery, personnel, method of concealment, informant(s), and modus operandi.
- Date: details time. Entries should follow a standardized format and be as detailed as possible. Fields include time, day, month, and year such as (e.g., mm-dd-yyyy hh:mm:ss)
- Place: details location the seizure occurred. These fields should provide detailed information about the specific area where the illicit products were detected. Fields include city, village, park, and coordinates (latitude and longitude).
- Trafficking Geography: information about product friction/flow and key sources and markets. This information should be as detailed as possible and include city names. The origin and destination ports, if known, should be specified. Fields include country of seizure, source, transit, and destination.
- Transit Routes: information about how the product was being transferred between the origin and destination. Fields include mode of transit (air, rail, multimodal), corporations/organizations involved, co-mingled goods, and path (direct, multi-stop).

#### Geospatial standards

Nearly all core categories in the data standards have an associated geospatial field (e.g., latitude/longitude, points/line/polygon, directional). The core categories, those critical for intersectional sharing, of the data descriptor and standards include:

- Conservation Information
  - Fauna or Flora: information about the specific products found in a seizure and their status/method of preparation. This information should be as detailed as possible, utilize standard units of measure, and avoid the use of abbreviations. Fields include dead (yes/no), wild (yes/no), commodity, species, classification, kingdom, phylum, quantity (kg., lb., count), and seizure phase (source, transit, destination).
  - Markets: details about the sale of illicit wildlife products. These fields should provide detailed information about the market location, conditions, and prices. Fields include conditions, price (units of sale), comingled goods, and vendor/seller.
- Criminogenic Information
  - Court: information identifying any court cases that arose from the seizure. Fields include case number, introduction date, closing date, judge, court, exact offense, predicate offense, people charged (number), and people fined (number).
  - Outcome: information about enforcement actions taken because of the seizure and whether they were successful. Fields include arrest (yes/no), arresting authority, type of charges, number of people, conviction (yes/no), type of charges (for conviction), acquitted (yes/no), and mutual legal assistance.
  - Sanctions: information about penalties levied on individuals caught participating in IWT. This information should be in standardized units of time and money. Fields include fine amount, fine currency, number of people fined, jail term, number of people jailed, and release dates.
  - Weapon: information surrounding the use of weapons in a specific case, including the specific weapon(s) used and any forensic evidence collected from them. Fields include weapon description, category (knife, gun, etc.), and forensics (caliber, DNA, financial).
- Data Integrity, Maintenance, and Interoperability
  - Report ID: the unique identifier for the report.
  - Reliability: provides a metric for measuring the value and quality of the information contained for the report. Fields include evaluation measures of the source, assessment of circumstances under which data was collected, and handling sensitivity.
  - Contact: links seizure record to a responsible individual who can answer questions about a specific incident and clarify data if needed. These fields should provide detailed information about how to contact the individual and their responsibilities/connection to the seizure *and* be protected under ethical guidelines that guarantee privacy and protection.

## Discussion

Geospatial data has supported design, implementation, and evaluation of interventions in diverse areas including public health, humanitarian relief, human rights documentation, and law enforcement^11–14^. We know from prior experience with these and other sectors that successful geospatial data standards need to be *useful* (i.e., identified by end users as important), *usable* (i.e., in a format with proper documentation/ metadata that allows for data to be properly ingested in any platform or system being used to manage information), and *used* (i.e., finds itself to an end user that can apply the knowledge to whatever sector in which they are operating). Geospatial information allows sectors working on IWT from a variety of disciplines to contextualize spatial relationships of crimes. Geospatial information can aid understanding about the spatial mobility of crime(s), offenders, and defenders; and be used to identify crime patterns, such as spatial-temporal clusters of activity. Law enforcement authorities can use such information to allocate resources for interdiction, conservation workers can more effectively target areas of concern for threatened populations, and researchers can more accurately describe wildlife trafficking supply chains and their dynamics. Geospatial information that captures movement along the entire supply chain—in both physical and virtual environments—can aid in determining the optimal location for interdiction activities, justice-oriented interventions, and allocating resources to regions where they are most likely to have an impact. Recording the location of activities surrounding the interdiction of wildlife trafficking can help enable multi-scale analysis of gaps in enforcement efforts and evaluation of wildlife protection and monitoring systems. Outputs could be integrated with other data analysis efforts and inform mechanisms for promoting communication, translation, privacy, and mediation across the knowledge-action boundary. Agencies and others can employ enterprise-specific privacy protection measures when sharing; enabling effective data collection in the first place is the paramount task. Pendleton *et al*.^8^. acknowledged the failure to move data from producers to users can lead to “data waste,” or lost opportunities to inform science and decision-making as well as result in costly replication of data collection efforts^1^. The data standards and dictionary presented here incorporate multiple data purviews and thus help converge scientific disciplines for decision-making, such as conservation, law enforcement and criminal justice, and supply chain sectors (Table 2). These sectors are not the only ones with relevance to IWT, but they were repeatedly mentioned by participants during the derivation of standards. Geospatial data in different formats (e.g., point, line, polygon) and at different scales can build understanding about, for example, wildlife species and populations of interest, targets of harm, and market prices. Organizations, agencies, researchers, and other sectors are expected to add additional fields and attributes depending on their mission and operational strategy, including for example, market characteristics, police reports and/or statistics, and location where sentencing occurs, but a foundational minimum enables cross-functional and cross-organizational compatibility and support.

**Table 2:** An example from United States District Court Southern District of New York, Sealed Indictment 19 CRIM 338 (United States of America v. Moazu Kromah, Amara Cherif, Mansur Surur, Abdi Ahmed).

Trafficking Geography	Fields, Subfields & Domain	Selected Example Seen in 19 CRIM 338	Nature of Spatialized Data (potential format)
Source	Country	Uganda, Democratic Republic of Congo, Guinea, Kenya, Liberia, Mozambique, Senegal, Tanzania	Counties where wildlife products originated and/or where the defendants originated to prove interstate commerce occurred (coordinates)
	Place	Cities where alleged offenders resided	(coordinates, time)
	Status	Dead	(attribute)
	Species Group	Black rhino, White rhino, African elephant (English and Latin names)	Forensic science by USFWS to prove which species was transported using interstate commerce (attribute)
	Number of Animals	35 rhinos, 100 elephants	Weight of rhino horn (190 kg) and weight of elephant ivory (10 tons) to prove degree of injurious wildlife provisions and inform penalties during sentencing (attribute)
Transit	Country	Senegal, United States	(coordinates)
	Place	Dakar, New York	Shipping rhino horn from Uganda to Dakar where it would be transported by others to Chinatown in New York helps prove interstate commerce, particularly import without a permit from USFWS (coordinates, time)
	Animal Status	Dead	(attribute)
	How Seized	Intercepted exchanged electronic messages including images, intercepted packages, telephone	(attribute, line)
	How Concealed	Pieces of African art such as masks and statues	(coordinates, attribute)
	Seizure Crime Scene 1	Package	(coordinates, attribute)
	Other Material	Narcotics, money laundering	10 kg of heroin, false real estate sale, concealed proceeds from sale of narcotics and wildlife in violation of the U.S. Lacey Act, and others (coordinates, polygon, attribute)
	Origin papers	Image of a shipping document concerning a particular package	Package was intercepted 13 days after image was shared, providing evidence of transport and documentation of lack of permits (coordinates, attribute)
Destination	Country	United States, Southeast Asia	(coordinates)
	Place	Manhattan	(coordinates, time)
	Number of Animal	1 black rhino horn, 2 white rhino horns	Photographs help provide evidence of the species in question (attribute)
	Who Collected	USFWS, DEA, law enforcement	Some US financial institutions involved with international wire transfers into foreign bank accounts, proving touchpoints to U.S. legal code(s) (attribute)
	Seizure Crime Scene	Narcotics, money laundering	Attempt to conduct financial transaction involving property to conceal proceeds from wildlife and narcotics (attribute, point, polygon)

Data ethics, integrity, and maintenance characteristics apply across all data fields. The potential format of geospatial data is italicized parenthetically in each applicability cell.

Geospatial data standards are important for combating IWT because the crime is intimately linked across space and time: knowledge about crime patterns and trends is required for crime prevention and response, including non-law enforcement-oriented response. Problem definition and solutions are fundamentally conceptualized in terms of location-based information: source (i.e., where does the killing and/or taking of wildlife take place?), transit (i.e., where, and how the wildlife and wildlife products are being moved along/through to market?), and destination (i.e., where are the wildlife consumers and where is wildlife marketed?). Geospatial data standards are a first step in integrating data that can be better linked to, for example, existing apps for mobile data collection or visualizations mapped results. The utility of tracking data across space and time is exemplified by research detailing illegal elephant ivory sampled from commercial markets and seized by authorities in airports^9,15^. The ability to apply a common set of geospatial information to link illegal wildlife products removed from various stages of the illicit supply chain back to the source location has enabled identification of crime hotspots, development of criminal profiles, indictments of alleged offenders, prosecutions in court, and indictments and convictions of kingpins (e.g., Table 1).

Standards in this data dictionary portend broad utility in support of investments to combat IWT, especially if standards can be mainstreamed. Substantial investments to combat IWT include capacity building activities, allocation of new financial and personnel resources, scientific discovery, regulatory changes, and public engagement activities. Monitoring these investments empowers stakeholders combating wildlife trafficking to evaluate progress and make evidence-based adjustments. How and whether use of standards and the relationship of data to confidentiality and access concerns is up to the community of practitioners, scientists, law enforcement authorities, and policymakers. The strength of geospatial data standards crimes not only from its content and structure but from the process by which it was created, in this case participatory, multisectoral, consensus-based, iterative, and interdisciplinary^16^. Local community stakeholders and expert practitioners were continuously engaged. There is intrinsic value in dissolving dataset boundaries that artificially constrict the necessary flow of information. For example, standardized geospatial data can foster transboundary engagement across geographies, institutions, and disciplines. As data availability increases the initial standard goal of interoperability may be broadened; the dictionary is publicly accessible online and any group can adapt it to their needs. The demonstrated value of geospatial data standards from other contexts (e.g., covid-19^17^, environmental models^18^ suggests the combating wildlife trafficking standards presented herein could support a similar positive impact, if the standards were adopted by as broad a range of stakeholders as possible. These could include, for example, non-governmental organizations with field operations (e.g., Chengeta Wildlife), inter-governmental organizations coordinating across geopolitical regions (e.g., Lusaka Agreement Task Force), and law enforcement authorities (e.g., Michigan Department of Natural Resources Office of Law Enforcement).

## Methods

Between 2017–2018, three workshops were convened on three continents with more than 160 individuals representing 80 organizations and institutions. Attendees represented multiple countries, cultures, and all were actively involved in documenting location-based observations about IWT^19^. Each workshop provided and encouraged multiple opportunities for attendees to interactively review the diversity of existing data collection efforts for IWT and collaboratively devise geospatial data standards^10^. The workshops’ “refine and revise” process for the standards was intentionally presented to participants as not having a built-in endpoint to allow for modifications, improvements, and ensure validity of the content^20^. The data dictionary was devised through a parallel iterative and engagement-based process at the three workshops:

- Workshop 1: On October 23, 2017, a half-day workshop was held at the Stimson Center in Washington, D.C. for approximately 60 individuals from different nongovernmental agencies, donor organizations, government agencies and institutions, universities, security firms, researchers, scientific societies, private sector companies and protected areas. Because this workshop was in Washington, D.C., participants were predominantly from government and NGO policy offices, but discussions were informed by field examples. Participants worked to answer questions such as what geospatial information would help end-users best combat IWT. They also answered how the sector could cope with “risky” data and conditions of delivery, what could be learned from other issues such as health, humanitarian crises, human rights, or convergent crimes, and identifying common database fields and metadata for geospatially enabled information in a transboundary context. Detailed insights from Workshop 1 including panel discussions, ideas for future action, and ideas for the data dictionary are available online^21^.
- Workshop 2: On March 26–28, 2018, a two-day workshop was held at the United Nations Economic Commission for Africa in Addis Ababa, Ethiopia for approximately 80 individuals from 22 countries. The same general diversity of participants was present as in the first workshop, though participants were by design predominantly from African-focused NGOs and most had GIS expertise. This workshop aimed to clarify the types and attributes of geospatial data most relevant for end users to support development of coordinated information databases. The workshop also sought to create new opportunities to realize benefits of standardization. Importantly, Workshop 2 was not convened with an explicit solution in mind a priori beyond leveraging geography to help achieve workshop objectives. Insights from Workshop 2 informed the first version of the draft data dictionary, which was built during the workshop and then revised collectively by all participants. The data standards were later presented electronically to the U.S. Interagency Task Force to Combat Wildlife Trafficking. The standards were posted and hosted on Esri’s Arc GIS Online site and participants from Workshop 2 were invited to use the standards and dictionary and provide feedback. Detailed insights from Workshop 2 including objectives, panel discussions, break out groups, and ideas for future action are available online^16^.
- Workshop 3: On October 22, 2018, a two-hour workshop was held as part of the 2018 Evidence to Action: Research to Address the Illegal Wildlife Trade event during the lead to the UK Government’s London Conference on the Illegal Wildlife Trade. Under the guidance of a facilitator, a group of approximately 30 scientists and NGO researchers working around the world with GIS on trafficking of wild fauna and flora worked collaboratively on laptop computers to review and revise the standards and dictionary, using. Participants built upon, discussed, and revised one another’s contributions to the standards through real time edits using Google Sheets. Given the “Evidence to Action” conference theme, participants were encouraged to prioritize effective use of evidence in decision-making as they edited. Revisions occurring during this workshop informed the final version of the standards and dictionary presented herein.

## Technical Validation

The need for technically validated geospatial data standards is broadly evidenced^22,23^. For example, the 2030 Agenda for Sustainable Development (¶ 76) noted the need for “…data systems to ensure access to high quality, timely, reliable and disaggregated data… including geospatial information^24^.” Geospatial data standards and descriptors can provide stakeholders with vetted attributes that allow IWT to be assessed within the goals-targets-indicator framework of the 17 Sustainable Development Goals (wildlife trafficking is relevant to at least SDG 8/Sustainable Economic Growth, SDG 14/Life below Water, SDG15/Life on Land, SDG 16/Peace, Justice & Strong Institutions). Another example is the United Nations’ General Assembly Resolution, “The Future We Want” (¶ 264,187) recognized “…the importance of *in situ* monitoring, knowledge and information sharing, and reliable geospatial information for sustainable development policy making^25^…” The U.S. Geospatial Data Act of 2018^26^ affirmed the need for geospatial data standards to improve environmental protection, economic development, and public health, mandating geospatial data standards be a) developed and promulgated (a priori and a posteriori) by voluntary standards consensus bodies; b) current, relevant, and effective; and c) electronically and publicly provided to harmonize sources and metadata.

Technical validation of the data standards occurred through presentations and discussions at professional society and scientific fora, including the (1) 4^th^ International Conference on Governance, Crime, and Justice Statistics. United Nations Office of Drugs and Crime. Lima, Peru, June 4–6, 2018; (2) Esri International User Conference in San Diego, California, July 9–13, 2018; (3) Asian Institute for Technology’s Bangkok Conference on Science, Technology, and Innovation for addressing Wildlife and Forest Crimes and Attaining SDGs, August 25–28, 2018; and (4) United States GEOINT Foundation Symposium 2021 on Discovery and Connections, October 5–8, 2021.

After the third workshop, the geospatial data standards and data dictionary were successfully deployed in the field to collect wildlife trafficking data across source, transit, and destination geographies (in Central African Republic, Democratic Republic of the Congo, and Republic of Congo) on species including elephants, pangolins, great apes, and dwarf crocodiles^27,28^. The standards also fit the recently unsealed indictment against four individuals accused of trafficking wildlife and heroin and laundering proceeds together in a real estate venture (e.g., Table 2). Spatialized data was assembled and used by authorities to identify potential criminal activity and potential criminal actors across the stages of the illicit supply chain (e.g., source, transit, destination). With calculated information, law enforcement authorities were able to communicate decision-quality data to criminal justice professionals who took prosecutorial action and conviction.

## Data Availability

The datasets generated during and/or analyzed during the current study are available at https://zenodo.org/badge/latestdoi/444480619 under a CC0 license^29^. There are no restrictions on access or use.

## Code availability

No custom code was used to generate the datasets or processes described in the manuscript. The process is available at the DOI providing repository https://zenodo.org/badge/latestdoi/444480619 with no restrictions on access or use.
